# Prediction of Pregnancy Complications With Maternal Biochemical Markers Used in Down Syndrome Screening

**DOI:** 10.7759/cureus.23115

**Published:** 2022-03-13

**Authors:** Savas Ozdemir, Orhan Sahin, Zuat Acar, Gozde Zeynep Demir, Ece Ermin, Alev Aydin

**Affiliations:** 1 Medicine and Surgery, Sisli Hamidiye Etfal Training & Research Hospital, Istanbul, TUR; 2 Obstetrics & Gynecology, Sisli Hamidiye Etfal Training & Research Hospital, Istanbul, TUR; 3 Perinatology, Sisli Hamidiye Etfal Training & Research Hospital, Istanbul, TUR

**Keywords:** beta-human chorionic gonadotropin (β-hcg), down syndrome serum screening tests, pregnancy outcome, papp-a, afp, maternal serum biomarkers

## Abstract

Objective: To determine whether first- and second-trimester maternal serum biomarkers are useful for the prediction of pregnancy complications like preterm birth, intrauterine growth restriction (IUGR), and macrosomia.

Methods: We conducted a retrospective analysis of 353 women having first- or second-trimester combined test for Down syndrome screening who delivered at our institution between January 2018 and December 2020. Associations between first- and second-trimester serum markers and adverse pregnancy outcomes among those who underwent prenatal screening for Down syndrome in our clinic were studied. The adverse pregnancy outcomes, serum levels of pregnancy-associated plasma protein-A (PAPP-A), β-human chorionic gonadotropin (β-hCG), and maternal serum alpha-fetoprotein (ms-AFP) were recorded and analyzed. Correlation analyses of PAPP-A, free βhCG, and ms-AFP with pregnancy outcomes were studied. We sought to predict the risks of preterm delivery (PTD, <37 weeks gestational age), low birth weight (LBW, <2500 grams) and macrosomia (>4000 grams).

Results: A total of 353 women who had first- and second-trimester screening test for Down syndrome were included. Two hundred fifty (70.08%) of them had first-trimester and 103 (41.2%) had second-trimester test. Mean age of the patients who underwent screening test for Down syndrome was 29.3±5.9, mean maternal weight was 67.3±13.6, mean gestational weeks at birth was 38.6±2.1 weeks and mean birth weight was 3260.9±511.1, preterm birth rate was 40/353 (11.3%), IUGR rate was 21/353 (5.9%), macrosomia rate was 17/353 (4.8%), stillbirth rate was 3/353 (0.8%). When laboratory and clinical parameters affecting birth weight and birth weeks were analysed in correlation analysis, both birth week and birth weight were found to be positively correlated with maternal weight. Of first-trimester markers Papp-A MoM (Multiples of Median) was found to be positively correlated with fetal birth weight (p = 0.044). Of second-trimester biochemical parameters ms-AFP was found to be negatively correlated with fetal birth weight (p = 0.039).

Conclusion: The study concluded that there is a relationship between serum markers and adverse pregnancy outcomes. Significant associations were found between the levels of first- and second-trimester serum markers PAPP-A, AFP and IUGR, macromia and additionally significant association was found between maternal weight and both delivery week and fetal weight. These results can highlight the pregnancies at risk and follow-up intervals may be arranged according to risk scala which may help at antenatal follow-up of high-risk patients.

## Introduction

Down syndrome (also known as Trisomy 21) is a genetic disorder that affects about 1/800 babies and causes both physical and mental health problems [1]. In babies with Down syndrome, there are three copies of chromosome 21, rather than two, or the specific area of chromosome 21 is modified as a result of translocation. It is the most common congenital cause of mental disability, and is associated with a number of congenital malformations in key organs, such as heart, kidneys, and gastrointestinal tract [1,2].

To detect this disorder in pregnancy, noninvasive screening tests based on biochemical analysis of maternal serum can be performed, and are used alongside fetal ultrasound measurements and maternal age to estimate the Down syndrome risk and trisomy 13/18 risk. When this screening test is done during the first trimester, it involves maternal age, nuchal translucency, and maternal serum biochemical analytes pregnancy-associated plasma protein-A (PAPP-A) and free β-human chorionic gonadotropin (fβ-hCG). On the other hand, second-trimester serum screening includes maternal age and weight, and maternal serum biochemical analytes, including alpha-fetoprotein (AFP), fβ-hCG and unconjugated estriol (uE3) or inhibin A, combined with gestational age and sonographic fetal biparietal diameter (BPD) measurements [3].

The combined first-trimester screening test can detect approximately 85% of Down syndrome cases with a 5% false-positive rate,while the second-trimester screening test yields an 81% detection rate and has a false-positive rate of 5% [3,4]. These biomarkers, however, have been shown to differ in adverse pregnancy outcomes, such as preterm delivery, fetuses that are small or large for gestational age, intrauterine growth restriction (IUGR), macrosomia, gestational hypertension, antepartum bleeding, etc. Moreover, because these biochemical markers are produced from feto-maternal placental interface, their levels could reflect placental diseases in the first and second trimester. Consequently, this gestation period is in focus of most extant studies [5,6]. The aim of the present investigation is to uncover the potential link between the maternal serum biochemical marker levels in the first- and second-trimester and pregnancy outcomes in our clinic.

## Materials and methods

In this retrospective study, medical records of 353 pregnant women that had singleton pregnancies and underwent the first-trimester (n = 250) or second-trimester (n = 103) biochemical screening tests at our maternal clinic between January 2018 and December 2020 were reviewed. Maternal age, gravidity, parity, gestational week at birth, fetal weight, and birth records were analyzed. Exclusion criteria included incomplete screening tests, giving birth at another center, fetal structural or chromosomal anomalies, and multiple pregnancies.

For first-trimester screening test, women had an ultrasound for NT and a blood sample taken for PAPP-A and free β-hCG between 11 weeks and 13 weeks 6 days of pregnancy, with an estimated risk detected on the basis of these combined markers and maternal age. Women undertaking second-trimester screening test had blood samples taken for the AFP, uE3 and f-βhCG measurements between 15 weeks and 20 weeks of gestation, which were combined with sonographic BPD measurements. The values of all serum markers were expressed as multiples of the median levels (MoM) of unaffected pregnancies at a given gestation [5]. The serum biomarker levels of all collected samples were determined at the same laboratory (completely automated assay, DELFIA® Xpress system; Perkin Elmer, Waltham, MA, USA), with the standard AFP, β-hCG, uE3, and PAPP-A immunoassay kits. Macrosomia is defined as birthweight that exceeds the 90th percentile for that gestational week, or ≥4,000 grams at term.

Premature birth is defined as any birth before 37 completed weeks of gestation, whereas small for gestational age (SGA)/IUGR is defined as <2,500 grams infant weight at term. SGA is often considered synonymous with IUGR, respectively defined as fetuses with birthweight below the 10th and 3rd percentile for the gestational age. IUGR is one of the main outcomes of high-risk pregnancy, and is associated with increased perinatal morbidity and mortality as well as abnormal neurodevelopment.

This study was conducted with the ethical approval of the Institutional Review Board (Prof. Dr. Cemil Taşcıoğlu Şehir Hospital, approval number 02/08/2021-292) and fully adhered to the Declaration of Helsinki principles. Informed consent was obtained from each research participant. The statistical procedures were undertaken using the Statistical Package for the Social Sciences (SPSS), Version 19.0 for Windows (SPSS Inc., IBM Corp., Armonk, NY, USA). For continuous variables mean ± SD was calculated, while n (%) was reported for categorical variables. The correlations between adverse pregnancy outcomes (IUGR, macrosomia, preterm labor) and maternal biomarkers (PAPP-A, β-hCG, AFP, and uE3) were determined by conducting Pearson correlation analysis, while logistic regression analysis was performed to determine the maternal weight cutoff.

## Results

During the study period, 353 women (mean age 29.3 ± 5.9 years, mean weight 67.3 ± 13.6 kg) underwent first-semester (250, 70.08%) and second-trimester (103, 41.2%) screening for Down syndrome at our clinic. The mean number of pregnancies was 2.0 ± 1.3, the mean gestational duration at birth was 38.6 ± 2.1 weeks, and the mean fetal birth weight was 3260.9 ± 511.1 grams., Our analyses revealed 40/353 (11.3%) preterm births, 21/353 (5.9%) cases of IUGR, 17/353 (4.8%) cases of macrosomia, and 3/353 (0.8%) stillbirths (Table 1).

**Table 1:** Demographic and biochemical variables PAPP-A: pregnancy-associated plasma protein-A, fẞhCG: free β-human chorionic gonadotropin, AFP: alpha-fetoprotein, uE3: unconjugated estriol, IUGR: intrauterine growth restriction, SGA: small for gestational age

Variables	Total number of patients (n=353) Mean±SD (min-max)
Age (year)	29.3±5.9 (17-44)
Weight (gr)	67.3±13.6 (39-120)
Gravida, n	2.0±1.3 (1-11)
Parite, n	0.5±0.7 (0-5)
First trimester test, n	250
Second trimester test, n	103
PAPP-A (mom)	1.07 (0.18-5.2)
fẞhCG (mom)	1.08 (0.25-4.82)
AFP (mom)	0.93 (0.4-2.19)
uE3 (mom)	0.89 (0.29-2.15)
Birthweek	38.6±2.1 (22-41.6)
Birth weight (gr)	3260.9±511.1 (400-4580)
Preterm delivery, n (%)	40/353 (11.3%)
Fetal Makrosomia, n (%)	17/353 (4.8%)
IUGR/SGA, n (%)	21/353 (5.9%)
Stillbirth,neonatal ex, n (%)	3/353 (0.8%)

When laboratory and clinical parameters affecting fetal birth weight and gestational age at birth were subjected to correlation analysis, these factors were found to be positively correlated with each other (p < 0.0001, r = 0.6452) as well as with maternal weight (at p = 0.036 and p < 0.0001, respectively). However, gestational age at birth was negatively correlated with maternal age (p < 0.0001, r = 0.6452).

As shown in Table 2, among the first- and second-trimester markers, only PAPP-A MoM was found to be positively and AFP was negatively correlated with fetal birth weight (at p = 0.044, r = 0.1272 and p = 0.039, r = - 0.2030, respectively). Maternal weight (mean = 67.3 ± 13.6 kg, range = 39−120 kg) was also found to be positively correlated with gestational age at birth. Moreover, logistic regression analysis revealed that maternal weight exceeding the mean (67 kg) was statistically significantly associated with the risk of fetal macrosomia (aOR = 5.5338, %95 CI = 1.5483−19.7781, p = 0.0034).

**Table 2:** Correlation analysis of clinic and biochemical markers with birth weight and birth week AFP: alpha-fetoprotein, HCG: human chorionic gonadotropin, fẞhCG: free β-human chorionic gonadotropin, PAPP-A: pregnancy-associated plasma protein-A, uE3: unconjugated estriol

Variables	Birth Week	Birth Weight
Maternal Age (yıl)	r= -0.1009 P=0.0583	r=0.08182 P=0.1250
Maternal Weight (gr)	r=0.1115 P=0.0366	r=0.2766 P<0.0001
Birth Week	1	r=0.6452 P<0.0001
Birth Weight (gr)	r=0.6452 P<0.0001	1
AFP	r = -0.1177 P=0.2364	r= - 0.2030 P=0.0398
AFP (mom)	r = - 0.08964 P=0.3678	r= -0.01860 P=0.8521
HCG	r=0.08628 P=0.3862	r= -0.06177 P=0.5354
HCG (mom)	r=0.1187 P=0.2323	r=0.01839 P=0.8537
uE3	r=-0.08359 P=0.4012	r=-0.1348 P=0.1745
uE3 (mom)	r=-0.1002 P=0.3140	r=-0.06695 P=0.5016
fẞHCG	r=-0.02360 P=0.7104	r=-0.08645 P=0.1730
fẞHCG (mom)	r=-0.01655 P=0.7946	r=-0.05082 P=0.4237
PAPP-A	r=0.01832 P=0.7732	r=0.06016 P=0.3435
PAPP-A (mom)	r=0.04413 P=0.4873	r=0.1272 P=0.0445

## Discussion

In extended research, maternal serum analytes used in the first- and second-trimester screening tests, such as PAPP-A and βhCG, have been found to be related to poor pregnancy outcomes, such as preterm labor, preeclampsia, antenatal bleeding, gestational diabetes, low birth weight, stillbirth, and fetal loss [5,7]. PAPP-A is produced by the placenta (in the syncytiotrophoblast cells) and is transmitted directly into maternal circulation, whereby its concentration increases as the pregnancy continues. PAPP-A is a protease for insulin-like growth factor (IGF) binding protein-4, as IGF regulates growth by controlling glucose and amino acid uptake in the trophoblast cells [6-8], it is obvious to expect that low PAPP-A values might be associated with growth disturbances like small or large for gestational age babies. Empirical evidence also indicates that PAPP-A changes in obstetric conditions that are linked to abnormal trophoblastic invasion in the first trimester (e.g., gestational hypertension, preeclampsia, fetal growth disturbances, preterm birth). Findings yielded by first-trimester screening tests further demonstrate that low PAPP-A levels are linked with an increased risk of pregnancy complications such as spontaneous fetal loss, low birth weight, IUGR, pregnancy-induced hypertension, preeclampsia, preterm birth, preterm rupture of membranes, and placental bleeding and abruption [8-10].

Guided by these findings, we examined the levels of these analytes in the pregnant women who gave birth at our clinic to uncover any associations with pregnancy complications. In our cohort, PAPP-A MoM was positively correlated with fetal birthweight (p = 0.044), as confirmed by other studies [7,9] linking low PAPP-A levels with low birthweight via insulin-like growth factors that regulate fetal growth. In addition to this, Spencer’s study [7] reported that the lowest PAPP-A levels were associated with the highest likelihood ratios for these complications. In this context, FASTER (First and Second Trimester Evaluation of Risk) trial findings are particularly noteworthy, as it included data on 34,271 pregnancies from multiple U.S. centers, suggesting that low PAPP-A levels are indicative of increased risk of spontaneous fetal loss, preterm birth, intrauterine growth retardation, gestational hypertension, and preeclampsia [11].

The second-trimester levels of maternal serum markers for aneuploidy (ms-AFP, βhCG, uE3, inhibin) have also been shown to be associated with adverse obstetric outcomes mostly due to disturbances in the placental function. Elevated AFP and hCG have been associated with low birthweight, IUGR, preeclampsia, as well as fetal stillbirth and preterm birth [12-14]. On the other hand, both lower and higher free βhCG values have been correlated with fetal loss and IUGR [5,15].

In the present study, only second-trimester msAFP values were found to be negatively correlated with fetal birthweight (p = 0.039), as confirmed by other authors [13,14]. These findings are expected, given that AFP is a glycoprotein that is produced from the second month of pregnancy by the yolk sac, and later in the fetal liver and gastrointestinal tract. AFP is then transported to maternal serum through the placenta or by diffusion across fetal membranes [15,16]. Elevated msAFP values are found to be associated with the fetal-maternal-placental barrier disruption, placental vascular damage or bleeding, or feto-placental ischemia and can compromise the development of a structurally normal fetus [16,17]. The relationship between the AFP increase in the second trimester and pregnancy complications (such as gestational hypertension, preeclampsia, fetal loss, preterm delivery, IUGR, placental abruption, and bleeding) has also been established [14-17]. Therefore, the link between AFP increase and IUGR yielded by our analyses is supported by empirical evidence [17]. It is also worth noting the link between low levels of msAFP (below 0.25 MoM) and spontaneous abortion, stillbirth, preterm birth, and macrosomia [14,15]. 

As hCG is produced by placenta, it is the first hormone detected in pregnancy [2,3]. However, at present, there is no consensus on the link between low first-trimester fβhCG levels and IUGR, preterm birth, and preeclampsia [11,12]. On the other hand, increased second-trimester hCG levels have been found to be associated with a number of adverse obstetric outcomes that are linked to placental dysfunction, such as preeclampsia, fetal loss, preterm delivery, and IUGR [11,18]. It is also worth noting the results reported by Chandra et al., who indicated that simultaneous elevation of AFP and hCG was a more reliable predictor of fetal death, preterm birth, IUGR, and pregnancy-induced hypertension compared to elevated values of one of these markers [19].

Given the growing incidence of overweight status and obesity in pregnancy, our finding that greater maternal weight was associated with greater fetal weight is relevant but an interesting finding in this study is increased maternal weight was associated with increased pregnancy duration. This is supported by the results reported by Halloran et al., who recommended restricting maternal weight gain in order to reduce fetal postmaturity [20].

A limitation of this study is that the study sample is small, so deriving strict conclusions is difficult, but similar studies we outlined above have large numbers and our results are consistent with them.

## Conclusions

Although abnormal first-trimester serum PAPP-A and second-trimester AFP levels were associated with adverse pregnancy outcomes in this study, the sensitivity of these tests is relatively low, limiting their clinical relevance. Nonetheless, these tests can be used to identify at-risk pregnancies and determine follow-up intervals that would allow adequate monitoring of both mother and the fetus.
